# The behavior of Tunisian students toward people with mental illness

**DOI:** 10.1192/j.eurpsy.2023.995

**Published:** 2023-07-19

**Authors:** M. Ben Amor, Y. Zgueb, A. Aissa, U. SchöBerlein Ouali, R. Zaibi Jomli

**Affiliations:** Psychiatry “A”, Razi Hospital, Manouba, Tunisia

## Abstract

**Introduction:**

Over the years, several studies have shown the high rate of discrimination experienced in particular by mental health service users. Stigma is composed of three elements: knowledge, behaviors, and attitudes. Although behaviors are the core of discrimination, this element has often been overlooked or intertwined with the other components.

**Objectives:**

Our study aimed to assess Tunisian students’ behavior toward people with mental illness

**Methods:**

This was a cross-sectional study conducted on 2501 Tunisian students who anonymously completed a form circulated online through social network groups and pages related to each academic institution. We have used the validated Arabic version of the “Reported and Intended Behaviour Scale” (RIBS) which assesses self-reported mental health behaviors and future intentions.

**Results:**

The median RIBS score was 15 out of 20, ranging from 4 to 20. Among the participants, 40% were living or have lived with someone with a mental health problem and 49.7% would be willing to live with someone with a mental health problem. Moreover, 24% were working or have worked with a person with a mental health problem and 53.4% would be willing to work with him or her. In addition, 34% were having or have had a neighbor with a mental illness and 58% would be willing to have a neighbor with a mental illness. Finally, 51% were having or have had a close friend with a mental health problem and 83.7% answered that they would be able to maintain a relationship with a friend who had developed a mental health problem.

**Conclusions:**

The assessment of behavior toward people with mental illness is fundamental as it has the most impact on individuals. However, behavior may be mediated by knowledge. Thus, it would be interesting to evaluate mental health knowledge to study the relationships between these constructs and optimize anti-stigma interventions.

**Disclosure of Interest:**

None Declared

